# Mid- to late-term follow-up of primary hip and knee arthroplasty: the UK SAFE evidence-based recommendations

**DOI:** 10.1302/2633-1462.42.BJO-2022-0149.R1

**Published:** 2023-02-09

**Authors:** Sarah R. Kingsbury, Lindsay K. K. Smith, Rafael Pinedo-Villanueva, Andrew Judge, Robert West, Judy M. Wright, Martin H. Stone, Philip G. Conaghan

**Affiliations:** 1 Leeds Institute of Rheumatic and Musculoskeletal Medicine, University of Leeds, Leeds, UK; 2 NIHR Leeds Biomedical Research Centre, Leeds, UK; 3 UK Faculty of Health & Applied Sciences, University of the West of England, Bristol, UK; 4 Nuffield Department of Orthopaedics, Rheumatology and Musculoskeletal Sciences, University of Oxford, Oxford, UK; 5 Translational Health Sciences, University of Bristol, Bristol, UK; 6 Centre for Statistics in Medicine, University of Oxford, Oxford, UK; 7 Tommy’s National Centre for Maternity Improvement, London, UK; 8 Leeds Institute of Health Sciences, University of Leeds, Leeds, UK; 9 Leeds Teaching Hospitals NHS Trust, Leeds, UK

**Keywords:** arthroplasty, hip, knee, follow-up, hip and knee arthroplasties, hip and knee arthroplasties, arthroplasty surgery, revision surgery, radiological evaluation, total hip and knee arthroplasty, orthopaedic surgeons, Hip, clinicians, Knee

## Abstract

**Aims:**

To review the evidence and reach consensus on recommendations for follow-up after total hip and knee arthroplasty.

**Methods:**

A programme of work was conducted, including: a systematic review of the clinical and cost-effectiveness literature; analysis of routine national datasets to identify pre-, peri-, and postoperative predictors of mid-to-late term revision; prospective data analyses from 560 patients to understand how patients present for revision surgery; qualitative interviews with NHS managers and orthopaedic surgeons; and health economic modelling. Finally, a consensus meeting considered all the work and agreed the final recommendations and research areas.

**Results:**

The UK poSt Arthroplasty Follow-up rEcommendations (UK SAFE) recommendations apply to post-primary hip and knee arthroplasty follow-up. The ten-year time point is based on a lack of robust evidence beyond ten years. The term 'complex cases' refers to individual patient and surgical factors that may increase the risk for arthroplasty failure. For Orthopaedic Data Evaluation Panel (ODEP) 10A* minimum implants, it is safe to disinvest in routine follow-up from one to ten years post-non-complex hip and knee arthroplasty provided there is rapid access to orthopaedic review. For ODEP 10A* minimum implants in complex cases, or non-ODEP 10A* minimum implants, periodic follow-up post-hip and knee arthroplasty may be required from one to ten years. At ten years post-hip and knee arthroplasty, clinical and radiological evaluation is recommended. After ten years post-hip and knee arthroplasty, frequency of further follow-up should be based on the ten-year assessment; ongoing rapid access to orthopaedic review is still required.

**Conclusion:**

Complex cases, implants not meeting the ODEP 10A* criteria, and follow-up after revision surgery are not covered by this recommendation.

Cite this article: *Bone Jt Open* 2023;4(2):72–78.

## Introduction

In 2019, over 100,000 hip arthroplasties plus a further 100,000 knee arthroplasties were carried out in the UK. The orthopaedic professional bodies traditionally advise follow-up of these patients at prescribed intervals.^1,2^ This places a significant pressure on the NHS orthopaedic services. If the existing recommendations on follow-up by the British Orthopaedic Association (BOA), British Hip Society (BHS), and British Association for Surgery of the Knee (BASK) were carried out using traditional out-patient follow-up appointments, then NHS orthopaedic services would be unable to see any new patients, as outpatient systems would reach full capacity with follow-up of joint arthroplasty patients. Various attempts have been made to cope with this problem in the UK. Some health authorities have moved to virtual clinic follow-up, while others have abandoned follow-up altogether.

Robert et al^3^ demonstrated that decommissioning is often about more than the ‘evidence’, and that withdrawal of previously available services is often seen as being driven by the wrong kind of evidence, based on cost data and political priorities and not on what patients and service users value. It is a complex issue, perhaps as contentious as National Institute for Health and Care Excellence (NICE) decisions when they do not recommend funding an effective intervention because it exceeds the cost-effectiveness threshold. However, NICE investment recommendations are made with the explicit understanding that, with no increase in the budget, there must be some displacement of other health care technologies.^4^

The UK poSt Arthroplasty Follow-up rEcommendations (UK SAFE) programme aimed to address the question: Is it safe to disinvest in mid- to late-term follow-up of hip and knee arthroplasty? Existing evidence demonstrates that early revisions are those that came with symptoms;^5^ therefore, the focus of the UK SAFE study was to fill the evidence gap for mid- to late-term follow-up where previous research evidence was lacking. For the purposes of this work, mid- to late-term follow-up was defined as more than five years post-primary surgery. A comprehensive evidence review was conducted, and additional research studies were undertaken where existing evidence was lacking. This included: a systematic review of the clinical and cost-effectiveness literature;^6^ analysis of routine national datasets to identify pre, peri- and postoperative predictors of mid- to late-term revision;^7,8^ collection and analyses of prospective data from 560 patients to understand how patients present for revision surgery;^9^ qualitative interviews with orthopaedic clinical leads and NHS service managers;^6^ and Markov modelling to simulate the survival, health-related quality of life, and NHS costs of patients following hip or knee arthroplasty surgery.^6-9^

This paper reports the recommendations from the final consensus process, which aimed to review the evidence gathered through the UK SAFE programme and obtain agreement for future care pathways to be recommended and adopted across the NHS.

## Methods

A consensus meeting was held in Liverpool, UK, on 12 September 2019. We made use of the recommendations for engagement and the use of evidence outlined in Robert et al^3^ to ensure the results of this work were understood and considered as a genuine attempt to use the best evidence available to ensure that the NHS gets value for money and that patients remain safe. We followed the methodological processes of NICE in developing the recommendations.^10^ These processes are based on internationally recognized standards, and are used by NICE for both their Technology Assessment Committees and Guideline Development Groups, to ensure that NICE recommendations provide the best possible evidence and guidance for NHS practice. NICE guideline recommendations use a wide range of different types of evidence, which is then reviewed and evaluated by a stakeholder committee, in order to develop the draft recommendations. The different types of evidence include the published literature, expert views, and patient experience.

Expert stakeholders with a special interest in patient follow-up after hip or knee arthroplasty surgery were invited to attend by distribution through the mailing lists of relevant professional bodies (including the BOA, BHS, and BASK) and special interest groups, through personal contacts and snowballing. Our aim was to ensure that all stakeholders were represented and balanced within the group. Many of the organizations were already aware of, and supportive of, the UK SAFE work, and therefore we asked these organizations to select an appropriate representative(s) for their organization to attend the meeting. In addition, we approached people that had clinical or research interests in this area; for example, the two GPs that attended both had a special interest in musculoskeletal (MSK) and both were involved in clinical commissioning group (CCG) work, including MSK pathway redesign. One GP was from the north of England and one from the South, therefore also providing geographical diversity.

Following the NICE consensus model, all participants received summaries of the main research findings in advance of the meeting, at which detailed presentations were given by the UK SAFE project team to outline the evidence for consideration. Following the presentations, consensus discussions took place until agreement was reached on the final recommendation statements.

## Results

A total of 32 stakeholders attended the final consensus meeting: patients (n = 5); general practitioners (GPs) (n = 2); representatives from major orthopaedic bodies (BHS (n = 4); BOA (n = 3); BASK (n = 2), Scottish Committee for Orthopaedics and Trauma (n = 1), Arthroplasty Care Practitioners Association (n = 2); National Joint Registry (NJR) (n = 1); ODEP (n = 3); NICE 2020 joint arthroplasty guideline committee (n = 1); Independent Healthcare Provider Network (n = 1); CCGs (n = 1); NHS England MSK (n = 1); implant manufacturers (n = 5); and 13 members of the UK SAFE project team.

It was agreed that recommendations should be grouped as overarching statements (to place the recommendations in context) followed by the recommendations themselves. These are presented below, together with summaries of the relevant discussion.

### Overarching statements

#### 1. These recommendations apply to post primary hip and knee arthroplasty follow-up

There was some general discussion about whether recommendations should be separated for hip and knee, or whether a single set of recommendations should be agreed to cover both hip and knee arthroplasty follow-up together. The consensus was that the evidence supported a single set of recommendations to cover both hip and knee arthroplasty. It was emphasized that these recommendations were for follow-up after primary surgery, and that they did not apply to follow-up after revision surgery.

There was agreement that any recommendations should provide scope for better ways of providing follow-up to be developed and tested, that face-to-face follow-up provision was not always necessary, and that innovations, such as virtual clinics and remote monitoring, could be incorporated into follow-up services.

The importance of educating patients to reduce the risk of the introduction of a rapid access service leading to additional/unnecessary costs through inappropriate self-referral was also highlighted. Education of both primary and secondary care clinicians was emphasized.

There was some general discussion about how disinvestment in follow-up may impact on disadvantaged groups, those hard to reach, and of low socioeconomic status. It was highlighted that the current evidence base misses those patients who are symptomatic but do not have appropriate follow-up, and therefore do not receive revision surgery despite needing it. It was agreed that further work was needed to understand how to reach such groups, and to explore the needs and outcomes in this population.

#### 2. The ten-year time point in these recommendations is based on a lack of robust evidence beyond ten years

There was agreement that the lack of available data beyond ten years of follow-up within the UK databases utilized to inform the evidence-base should be noted, and that a recommendation to disinvest in follow-up beyond ten years could not be supported.

#### 3. In these recommendations, the term complex cases refers to individual patient and surgical factors that may increase the risk for arthroplasty failure

In addition to discussion around prosthesis rating, as highlighted below, there was agreement that additional factors must be considered when determining whether a patient required additional follow-up provision. Age should be relevant in clinical review, with younger patients more likely to have a failing implant, while older patients are less likely to out-live their prosthesis. Surgical experience may be important. For junior surgeons, follow-up may provide some additional benefit with respect to their own training and development. Additional surgical factors and patient demographics may also increase the risk for arthroplasty failure, and these factors should be considered prior to disinvestment in follow-up for an individual patient.

### Recommendations

#### 1. For Orthopaedic Data Evaluation Panel (ODEP) 10A* minimum implants, it is safe to disinvest in routine follow-up from one to ten years post-non-complex hip and knee arthroplasty, provided there is rapid access to orthopaedic review

There was general agreement among surgeons that, based on the evidence from UK SAFE and from their own clinical experience, for a routine patient with an ODEP 10A* prosthesis,^11^ they would be happy to discharge after the six-week postoperative check and not see the patient again until ten years. With modern on-the-shelf revision implants and surgical techniques of allografting and impaction grafting, there is often less urgency to proceed to revision surgery for asymptomatic radiological changes than there was ten years ago. Even if detected, cases are now commonly kept under observation, with development of symptoms the trigger to proceed to surgery. However, it was recognized that some surgeons or units may wish to retain the one-year follow-up. It was agreed that we could not currently state that follow-up ‘is not needed’, but that there was sufficient evidence to state that it was ‘safe to disinvest’. However, there was much emphasis on the need for a rapid access service to orthopaedics to ensure that patients could access support if the need arose. Surgeons also highlighted the importance of ensuring that these recommendations did not enable NHS trusts to completely disinvest from all follow-up, with no safety net for patients. Abandoning all follow-up facilities for these patients should not be part of the recommendation from this study. Rather than complete disinvestment in all follow-up up to ten years, the UK SAFE guidelines should require the provision of different follow-up facilities for these patients. It is likely that the new facilities will be cheaper to provide than the current ones. No changes in follow-up arrangements should be made unless a pathway is available for urgent review, ideally straight into secondary care, for patients with hip or knee arthroplasties who develop new symptoms in their operated joint. Patients agreed that the key to ensuring that disinvesting in follow-up was safe, was to ensure that all patients had access to a robust, simple, and safe mechanism for re-accessing orthopaedic support. GPs highlighted that referral into a rapid access system could be initiated by a GP, but that patient-initiated self-referral could also be considered.

The difficulty of setting up an efficient, cost-effective rapid access clinic was highlighted, since this can be difficult to plan to ensure rapid availability to appointments without the risk of leaving empty clinic slots. This work does not support disinvestment in the follow-up service without such a rapid access service being available for, and direct access by, the patients. The need for further work to understand how these would work was emphasized.

#### 2. For ODEP 10A* minimum implants in complex cases, or non-ODEP 10A* minimum implants, periodic follow-up post hip and knee arthroplasty may be required from one to ten years

Surgeons and GPs highlighted that any recommendations must qualify that ‘safe disinvestment of follow-up’ only applies to those prostheses that are endorsed by the existing recommendations, such as those from ODEP, NJR, and the Medicines and Healthcare products Regulatory Agency (MHRA). Any that do not meet these standards may require additional follow-up, and this must be stipulated and considered on a prosthesis-specific basis. One industry representative highlighted some concern that this was more complex for knee than for hip, due to ODEP ratings being based upon multiple construct factors, which could lead to difficulty in classifying patients for follow-up/no follow-up. The use of current NJR data would be essential when identifying combinations of prostheses that require additional follow-up. We recommend that the lowest ODEP rating of all the components of the joint should determine the overall ODEP rating of that joint arthroplasty.

In addition, it was emphasized that, in some cases, it is the need of the patient that should drive follow-up and not just the prosthesis, and for complex cases or patients with complex needs, then more regular follow-up must also be considered. For example, very young patients may be at an increased risk of revision surgery, while other potential risk factors include, but are not limited to, comorbidities, and pre- and postoperative pain and function.^7,8,12,13^

#### 3. At ten years post-hip and knee arthroplasty, clinical and radiological evaluation is recommended

Following on from discussion regarding the lack of current data to support disinvestment beyond ten years, stakeholders agreed that all patients should be given the opportunity to re-present for review of their joint arthroplasty. There was emphasis from most surgeons that this review must include both clinical and radiological review, since issues such as silent osteolysis, which become more common after ten years, may be missed by clinical/patient-reported review alone. There was support for the potential use of virtual clinics for such review, provided clinical and radiological review were incorporated.

#### 4. After ten years post-hip and knee arthroplasty, frequency of further follow-up should be based on the ten-year assessment; ongoing rapid access to orthopaedic review is still required

There was general agreement that follow-up beyond ten years should be based on the clinical and radiological review at the ten-year time point, but that continued rapid access to orthopaedic review, if necessary, should be re-emphasized.

### Key areas for further research in hip and knee arthroplasty follow-up

Key areas for further research were agreed at the consensus meeting, as outlined in Table I.

**Table I.** Future work.

1. Further work is recommended to review the data on care of patients with joint arthroplasty beyond ten-year follow-up. At the present time robust recommendations cannot be made due to lack of robust data beyond ten years of follow-up. Further study of the revisions beyond ten years is suggested to see if the time period to asymptomatic review can be extended.
2. A study of the different local models of follow-up based on these UK SAFE recommendations will provide information on the success and cost of these models once adopted.
3. A comparison of areas with no follow-up and the UK SAFE follow-up model will give insights as to the benefit of regular, > ten-year, follow-up for these patients. A cost-to-benefit study of these models would advise on the next model of follow-up. Data from no follow-up from the UK SAFE study could be used in a future comparison study.
4. Further work is needed to establish the most effective model of delivering a rapid access service.
5. Extrapolation and evaluations of this pathway for other joints may prove cost-effective, and beneficial for patients and their surgeons. Approach and involvement of the appropriate specialist societies would be required to extrapolate and develop these recommendations further into other joint arthroplasties.
6. Disinvestment in follow-up may impact on disadvantaged groups, those hard to reach and of low socioeconomic status. The current evidence base misses those patients who are symptomatic but do not have appropriate follow-up and therefore do not receive revision surgery despite needing it. It was agreed that further work was needed to understand how to reach such groups, and to explore the needs and outcomes in this population.
7. Virtual clinic models have previously been evaluated for hip and knee arthroplasty follow-up,^14-16^ and are already established in some centres. The COVID-19 pandemic has led to a proliferation of virtual clinics and further work is needed to evaluate different virtual models and to understand how patient self-referral may be integrated into a virtual clinic service. The virtual clinic would then evolve into a long-term UK SAFE follow-up pathway for the patient.
8. Further work is needed to examine how patient specific outcome scores can help in predicting long-term risk of prosthetic failure in the context of quality of life.
9. Further exploration of the factors identified as increasing risk of revision and which may contribute to case ‘complexity’, for example preoperative pain medication and implant factors.

## Discussion

The UK SAFE study has demonstrated that for ODEP 10A* prostheses, it is safe to disinvest in routine follow-up in the one- to ten-year period after non-complex total hip and knee arthroplasty. At ten years after index surgery, clinical and radiological review is recommended. Complex cases, implants not meeting the ODEP 10A* criteria, metal-on-metal implants, and follow-up after revision surgery are not covered by this recommendation. Omission of recommendation for disinvestment of follow-up beyond ten years is based on the absence of robust data to either support or refute the same advice beyond the initial ten-year follow-up period. Determining the optimal way to conduct long-term models of follow-up was beyond the scope of UK SAFE.

These recommendations have potential for major impact on how, where, and when patients with hip and knee arthroplasties are followed-up, especially in the post-COVID-19 era and in view of the current national agenda for speciality redesign and optimization of outpatient care, increased used of remote monitoring, and the move to personalize care provision. Once the patient has completed the routine joint arthroplasty follow-up at, for example, three months, no further follow-up or radiograph is required at one year, seven years, or before ten years, when a follow-up with radiograph is recommended. All routine follow-up appointments arranged for between one and ten years should be cancelled. The patients should attend for a ten-year follow-up appointment as per the current model of follow-up in their area. The impact will be to reduce the burden on both patients and the NHS in terms of outpatient visits and clinical tests that do not add benefit, while enabling resources to be focused on optimizing detection of potential problems.

We did not study how or where future follow-up services will be run, or the cost-effectiveness of such alternative models of follow-up. However, before abandoning current follow-up services and moving patients to a new service, several requirements should be considered (Table II). With the planned setting up of UK regional joint arthroplasty revision services (hub and spoke model), this pathway will become an essential part of this new revision service. Follow-up of hip and knee arthroplasty patients may be virtual, involving patient-reported and radiological review. Virtual clinic models have previously been developed and evaluated for hip and knee arthroplasty follow-up and are already established in some centres.^14,15^ Each major regional centre should identify a radiograph facility with the appropriate expertise to offer this service. This may be run by the radiology department, orthopaedic department, or a combined multi-disciplinary team. The expertise in interpreting joint arthroplasty x-rays is more important than who runs this service. The specialist societies may consider validating/approving these centres. Evidence-based standardized radiology reporting should be considered, as, for example, that previously developed by members of the UK SAFE team.^14^ A local information leaflet, paper or on-line, and in multiple languages, should detail the service provision for these patients. Further details of how to access the system and red flags for the patients should be listed.

**Table II.** Suggestions as to how a follow-up service based on the UK SAFE recommendations might work.

1. Patients should be empowered to share or take control of their own follow-up after hip or knee arthroplasty.
2. Patients should be provided with written details of their implant and its ODEP rating. Only ODEP 10A* and above are suitable for the new model of follow-up.
3. Patients should be asked if they are willing to provide consent to their data being collected centrally on a national database.
4. Patients should be provided with written instructions as to the timing of their next review with x-rays. A login and personal password could be provided to a local or national online follow-up joint arthroplasty pathway web site.
5. GPs should be provided with details of the model of follow-up, when the next radiograph or follow-up is due to take place and how to access the rapid access system if required.
6. Where possible, the patient should have access to self-referral to a local virtual clinic accepting that this may or may not be the secondary care where the primary surgery was carried out. Strict screening and triage criteria will need to be in place for this.
7. This self-referral may be through an on-line portal or directly with their local provider.
8. Secondary care should develop an approved and accredited radiograph follow-up service, which may be virtual, for GP referral or patient self-referral should a patient develop pain in, or problems with, one of their replaced joints. The approved radiograph service should have a special interest in joint arthroplasty review with a lead radiologist who has a special interest in joint arthroplasty follow-up.
9. If a patient finds themselves in an area without a UK SAFE pathway in place when they develop pain or other problems with their joint arthroplasty, then urgent referral to a secondary hip or knee arthroplasty service should be made. An radiograph of the joint should be arranged immediately if an appropriate follow-up appointment is not available or if there is concern regarding impending fracture around the implant. The radiograph should be reviewed by a member of the orthopaedic team for impending problems and a decision on appropriate action. Depending on the local service this patient may then be treated locally or referred to the tertiary hub for revision surgery in that region. Patients with systemic symptoms should be referred urgently, without starting any antibiotics.
10. If the above 1 to 9 are in place, then it is safe to disinvest in routine follow-up before ten years after hip and knee arthroplasty surgery.

ODEP, Orthopaedic Data Evaluation Panel.

Patient and clinician education is also important when considering the implementation of new and revised pathways. Both patients and clinicians who will have their follow-up arrangements changed by these recommendations will require explanation, education, and training. The exact methods used may differ from region to region depending on local facilities available. Education and ownership by patients are key to success in the roll-out of these new services. Local Patient and Public Involvement (PPI) groups, interested GPs, and secondary care teams should be involved in the planning of these services. Patients must be empowered to take responsibility for their care, with routes for them to take action when needed available, aligning with the long term approach to enhance patient initiated follow-up across the NHS.

There were limitations to this work. There was a lack of sufficient data beyond ten years of follow-up within the UK databases used to inform the evidence-base, and consequently a recommendation to disinvest in follow-up beyond ten years could not be supported.

In conclusion, the UK SAFE programme demonstrated that for ODEP 10A* prostheses, it is safe to disinvest in routine follow-up in the one- to ten-year period after non-complex hip and knee arthroplasty. At ten years after index surgery, clinical and radiological review is recommended. Complex cases, implants not meeting the ODEP 10A* criteria, metal-on-metal implants, and follow-up after revision surgery are not covered by this recommendation.

Recent NICE guidelines on hip, knee, and shoulder arthroplasty (NG157)^17^ stated that the committee were unable to make recommendations on follow-up due to a lack of evidence in this area. The results of the UK SAFE study provide some of the missing evidence, although there is a need for further research, as detailed in the NICE guidelines.

**Take home message**

- Previous advice on review at one, seven, and ten years are superceded by these evidence-based recommendations.

- Clinicians should consider how local referral pathways can facilitate rapid access to orthopaedic review for hip and knee arthroplasty patients.

## Data Availability

The datasets generated and analyzed in the current study are not publicly available due to data protection regulations. Access to data is limited to the researchers who have obtained permission for data processing. Further inquiries can be made to the corresponding author.
